# Cripowellins Pause *Plasmodium falciparum* Intraerythrocytic Development at the Ring Stage

**DOI:** 10.3390/molecules28062600

**Published:** 2023-03-13

**Authors:** Joshua H. Butler, Heather J. Painter, Emily K. Bremers, Priscilla Krai, Manuel Llinás, Maria B. Cassera

**Affiliations:** 1 Department of Biochemistry and Molecular Biology, University of Georgia, Athens, GA 30602, USA; 2 Center for Tropical and Emerging Global Diseases (CTEGD), University of Georgia, Athens, GA 30602, USA; 3 Division of Bacterial, Parasitic, and Allergenic Products, Office of Vaccines Research and Review, Center for Biologics Evaluations and Research, Food and Drug Administration, Silver Spring, MD 20993, USA; 4 Department of Biochemistry, Virginia Tech, Blacksburg, VA 24060, USA; 5 Department of Biochemistry and Molecular Biology, The Pennsylvania State University, University Park, PA 16802, USA; 6 Huck Center for Malaria Research, The Pennsylvania State University, University Park, PA 16802, USA; 7 Department of Chemistry, The Pennsylvania State University, University Park, PA 16802, USA

**Keywords:** *Plasmodium*, *Crinum erubescens*, cripowellin, ring stage, cytostasis, transcription

## Abstract

Cripowellins from *Crinum erubescens* are known pesticidal and have potent antiplasmodial activity. To gain mechanistic insights to this class of natural products, studies to determine the timing of action of cripowellins within the asexual intraerythrocytic cycle of *Plasmodium falciparum* were performed and led to the observation that this class of natural products induced reversible cytostasis in the ring stage within the first 24 h of treatment. The transcriptional program necessary for *P. falciparum* to progress through the asexual intraerythrocytic life cycle is well characterized. Whole transcriptome abundance analysis showed that cripowellin B “pauses” the transcriptional program necessary to progress through the intraerythrocytic life cycle coinciding with the lack of morphological progression of drug treated parasites. In addition, cripowellin B-treated parasites re-enter transcriptional progression after treatment was removed. This study highlights the use of cripowellins as chemical probes to reveal new aspects of cell cycle progression of the asexual ring stage of *P. falciparum* which could be leveraged for the generation of future antimalarial therapeutics.

## 1. Introduction

**Figure 1:**
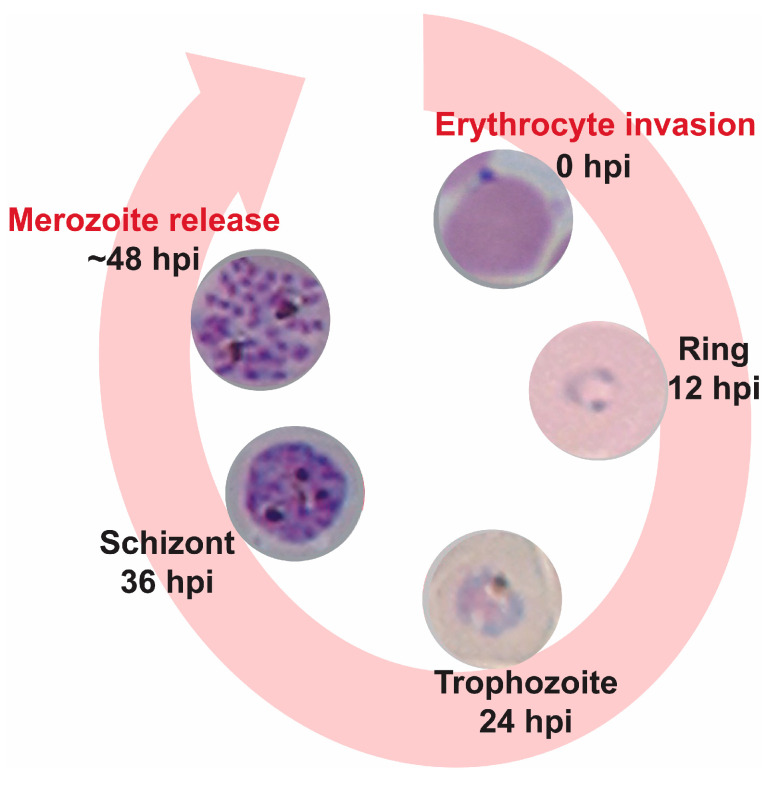
The asexual intraerythrocytic developmental cycle (IDC) of *P. falciparum.* The cycle occurs over 48 h, beginning when a merozoite invades an uninfected red blood cell. After invasion, merozoites develop progressing through the ring, trophozoite, and multinucleated schizont stages, ending with the formation and egress of merozoites that will initiate a new cycle after invasion of a new red blood cell; hpi: hours post-infection.

Malaria remains a serious parasitic disease in the world. In 2021, over 247 million new infections and 619,000 deaths were reported, mostly caused by *Plasmodium falciparum* [1]. The asexual intraerythrocytic developmental cycle (IDC) of *P. falciparum*, which is responsible for the clinical manifestation of malaria, typically occurs over the course of 48 h beginning when a merozoite invades an uninfected red blood cell (Figure 1). After invasion, merozoites progress through the ring and trophozoite stages before undergoing schizogony, generating new nuclei through asynchronous rounds of genome replication prior to cytokinesis. Newly formed merozoites (~16–32) egress and infect new red blood cells, thus beginning a new cycle. With a few exceptions, such as artemisinin and cipargamin (KAE609), the majority of antimalarial drugs target maturing trophozoite and schizont stages in the IDC, where the parasite performs essential—and druggable—metabolic and cellular processes, such as hemoglobin digestion, mitochondrial and apicoplast function and development, as well as DNA and RNA synthesis [2,3]. However, antimalarial drugs targeting the *P. falciparum* ring stage are highly attractive as they can prevent the development of the trophozoite and schizont stages that are sequestered by cytoadherence of infected erythrocytes to the endothelial cells of deep vascular beds in vital organs. In addition, the ring stage precedes gametocytogenesis, the intraerythrocytic sexual development stage required for transmission of the parasite to the *Anopheles* mosquito vector, thus reducing or blocking the transmission of the disease [4,5].

From a molecular perspective, how the malaria parasite regulates the cell cycle and the timing of each intra-erythrocytic stage is currently not well understood [6]. The progression from ring to the trophozoite stage in the IDC has previously been shown to be susceptible to perturbation via nutrient deprivation of polyamines and amino acids [7,8,9,10,11], and is also a hallmark of artemisinin-induced ring stage dormancy in response to drug treatment [12,13,14,15]. Some of these perturbations have been shown to be reversible by exogenous supplementation of depleted metabolites, the removal of the small molecule inducing the phenotype, or a combination of both [16].

Natural products have made the greatest contribution to the treatment of malaria as well as providing structural inspiration for the development of new antimalarial drugs. The sweet sagewort plant named Qinghao (*Artemisia annua*) was used as early as the second century BCE in China to treat intermittent fever [17]. Structural derivatives of artemisinin, the active component of *Artemisia* extracts, in combination with partner drugs known as artemisinin-based combination therapies (ACTs), are the current frontline therapies for treating malaria [18]. In the past decade, the antimalarial activities of over 1500 natural products have been reported [19]. However, their complex structures and limited synthetic availability have hampered the evaluation of their mechanisms of action as well as pharmacological properties. Natural products are likely to have novel molecular targets due to their structural uniqueness and complexities guaranteeing their sustained exploration as antimalarial agents as well as biological probes.

The genus *Crinum* belongs to the family Amaryllidaceae and includes around 110 accepted species. Members of the genus *Crinum* have umbels of lily-like flowers on leafless stems and are found in moist sites, including forests, marshes, and swamps, and along the sides of streams and lakes in tropical and subtropical areas worldwide [20]. Extracts from *Crinum* species have been used traditionally to treat a variety of illnesses including fever, swelling, wounds, cancer, and malaria [21]. The *Crinum* genus is known to be a rich source of alkaloids, including lycorine, crinine, and narciclasine. Over 650 alkaloids have been extracted from Amaryllidaceae bulbous plants and have shown a wide range of biological potentials including anticancer, antibacterial, antiviral, antifungal, and antimalarial activity [22].

Our previous work on *Crinum erubescens* L. f. ex Aiton to search for novel antiplasmodial agents from plants, led to the isolation of two known pesticidal compounds cripowellin A and B, and two new compounds cripowellin C and D [23]. The unusual amaryllidaceae alkaloids cripowellins A and B were originally isolated from *Crinum powellii* [24]. All four compounds have potent antiplasmodial activity with half-maximal effective concentrations (EC_50_) ranging from 26 to 260 nM, compared to artemisinin (6 nM) used as control [23]. Unfortunately, due to their complex structures and lack of complete synthetic availability, the evaluation of their mechanisms of action have been hampered [25,26]. As shown in Figure 1, the malaria parasite undergoes a complex ~48 h developmental cycle with substantial morphological changes that are concurrent to equally extensive shifts in biological processes at the molecular level where the action of antimalarials is constrained by these temporal dynamics. Phenotypic drug assessments in the IDC are usually performed in synchronous cultures with continuous drug exposures of 72 h. During this time, parasites have undergone a round of replication and reinvasion, therefore, the specific stages being targeted by compounds cannot be determined. Stage-specific susceptibility of *P. falciparum* asexual blood stage parasites is a key step in studying how compounds may kill the malaria parasite [2]. In this report, studies to determine the timing of action of cripowellins within the IDC led us to the observation that this class of natural products induced reversible cytostasis in the ring stage. However, in the trophozoite and schizont stages they are cytotoxic. In addition to the pesticidal activity, cripowellins are also toxic to mammalian cancer cell lines with EC_50_ ranging from 11 to 28 nM [25,27], therefore, we consider this class of compounds as chemical probes rather than potential antimalarials. This study highlights the use of cripowellins as chemical probes to reveal new aspects of cell cycle progression of the asexual ring stage of *P. falciparum* which could be leveraged for the generation of future antimalarial therapeutics.

## 2. Results and Discussion

### 2.1. Cripowellins A and B Pause Parasites in Rings but Kill Trophozoite and Schizont Stages

**Figure 2:**
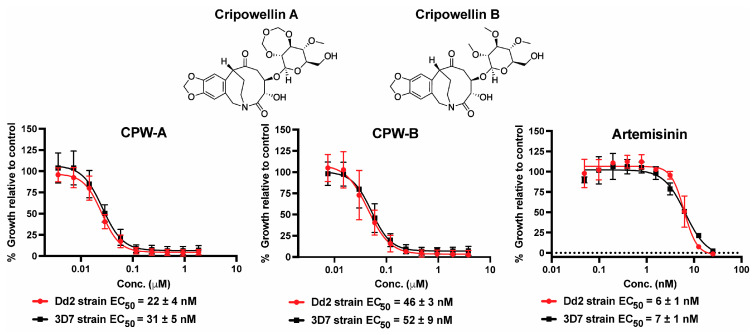
In vitro potency of cripowellin A (CPW-A) and cripowellin B (CPW-B) in *P. falciparum* drug-sensitive (3D7) and drug-resistant (Dd2) strains. Artemisinin was used as control. The EC_50_ reported values represent averages and the SEM of three independent assays.

The potency of cripowellin A (CPW-A) and cripowellin B (CPW-B) was first re-evaluated to confirm their activity against the *P. falciparum* Dd2 strain (resistant to chloroquine, pyrimethamine, and mefloquine) using an established SYBR Green I endpoint assay after 72 h of continuous drug exposure (Figure 2) [23,28]. The potency of CPW-A and CPW-B was also tested against the susceptible 3D7 strain and similar EC_50_ values were obtained suggesting that both compounds are apparently not subject to the resistance mechanisms of chloroquine, mefloquine, and pyrimethamine. A limitation of this approach is that it only evaluates the increase in the DNA content as an indirect measure of growth and cannot differentiate between cytostatic and cytocidal antimalarial mechanisms of action.

**Figure 3:**
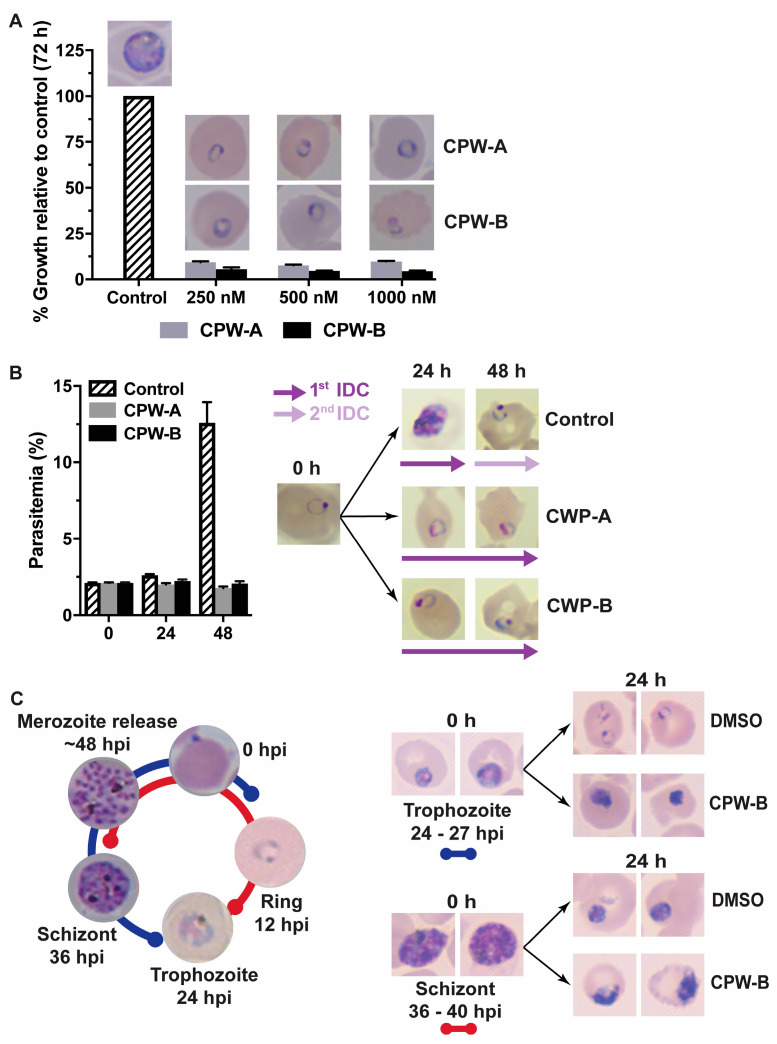
CPW-A and CPW-B pause asexual IDC at ring stage in *P. falciparum*. (**A**) Inhibition of growth was determined by the SYBR Green I assay after 72 h of continuous exposure to CPW-A and CPW-B. Values are reported as percentages relative to untreated controls. A representative Giemsa-stained thin blood smear is shown. (**B**) Parasitemia was determined by light microscopy counting of *P. falciparum*-infected erythrocytes treated with either DMSO (0.01%, control), 500 nM CPW-A, or 500 nM CPW-B. A representative Giemsa-stained thin blood smear is shown for cultures before treatments started (0 h) and at the indicated times. The reported values represent averages and the SEM of three independent assays. (**C**) Highly synchronous trophozoite or schizont stage cultures at 2% parasitemia were treated with 500 nM of CPW-B for 24 h. A representative Giemsa-stained thin blood smear is shown before treatment started and after 24 h of continuous drug exposure.

Therefore, to investigate the antimalarial mechanism of action of CPW-A and CPW-B, we assessed their potential effects on the asexual intraerythrocytic stages and morphological development by Giemsa-stained thin blood smears and light microscopy. Highly synchronous ring stage cultures (8–12 h post-infection, hpi) were treated with CPW-A or CPW-B at concentrations indicative of near complete growth inhibition of (250, 500, and 1000 nM) for 72 h, at which time Giemsa-stained thin blood smears of treated and control untreated parasites were collected, and growth was measured by the SYBR Green I assay (Figure 3A).

Interestingly, morphologically normal ring stage parasites were observed in smears from all three concentrations tested where low growth was detected by assessing DNA content. Since treatment was started at the ring stage in highly synchronous cultures, parasites could have egressed and reinvaded after 72 h (Figure 1). Therefore, it is possible that the observed ring stage parasites were either from the first or second IDC. To differentiate between these two possibilities, ring stage parasites (8–12 hpi) were treated with 500 nM of CPW-A or CPW-B, and both parasitemia and morphology were assessed at 24 and 48 h by Giemsa-stained thin blood smears and light microscopy (Figure 3B). Interestingly, cultures treated with CPW-A or CPW-B remained at the 2% initial parasitemia over the course of 48 h, while control parasites completed one IDC and re-invaded red blood cells over the same time period. Morphological assessment of treated parasites revealed that both CPW-A and CPW-B appear to arrest the progression of the IDC halting parasites at the ring stage. On the other hand, a cytotoxic effect was observed when highly synchronous trophozoite or schizont stage cultures were treated with 500 nM of CPW-B for 24 h (Figure 3C). Taken together, these results confirmed that CPW-A and CPW-B treatments pause progression of the IDC in the ring but not in trophozoite and schizont stages.

### 2.2. Cripowellin B Is Cytostatic in Ring Stage Parasites

**Figure 4:**
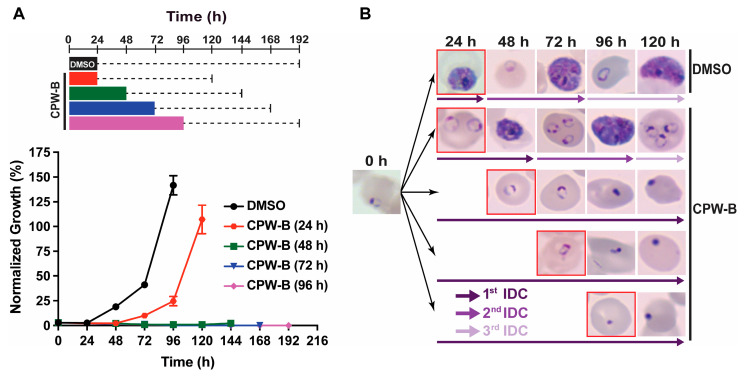
CPW-B is cytostatic in ring stage parasites after 24 h of treatment. (**A**) *P. falciparum* 3D7 strain cultures were treated with 500 nM CPW-B for 24 h (red), 48 h (green), 72 h (blue), 96 h (purple) or 0.01% DMSO (black) for 24 h as shown in the scheme. At the indicated time CPW-B was washed out and cultures were maintained in drug-free media (dashed line). Parasitemia was monitored every 24 h by Giemsa-stained thin blood smear and light microscopy. Normalized growth was calculated by multiplying the parasitemia value by the dilution factor for subculturing to 2% parasitemia every 48 h in actively growing cultures. The reported values represent averages and the SEM of three independent assays. (**B**) A representative Giemsa-stained thin blood smear is shown for each condition tested. Red squares indicate that CPW-B was washed out and parasites were returned to culture in the absence of drug.

To further investigate this phenomenon, CPW-B was selected as sufficient quantities were available. To assess if parasites would recover from treatment with CPW-B, highly synchronous ring stage parasites were treated with 500 nM CPW-B for 24, 48, 72, and 96 h. At each indicated time, CPW-B was washed out and parasites were maintained in drug free media for an additional 96 h. Parasitemia and intraerythrocytic stage progression were monitored over time by Giemsa-stained thin blood smears and light microscopy (Figure 4). Parasites exposed to CPW-B for 24 h resumed normal asexual IDC and growth after CPW-B was washed out as evidenced by the observation of schizonts at 48 h after the experiment was initiated and appearance of ring stage parasites at 72 h (Figure 4B).

Interestingly, while parasites treated continuously with 500 nM of CPW-B for 48 and 72 h remained in the ring stage, treatment ultimately prevented parasites from resuming asexual stage progression following washout of CPW-B and died. Therefore, these observations provide evidence that treatment with CPW-B for 24 h results in a cytostatic “pausing” event in ring stage parasites while longer treatments are cytocidal to the parasite, stopping the asexual IDC.

### 2.3. Cripowellin B Pauses Transcriptional Progression through the IDC

**Figure 5:**
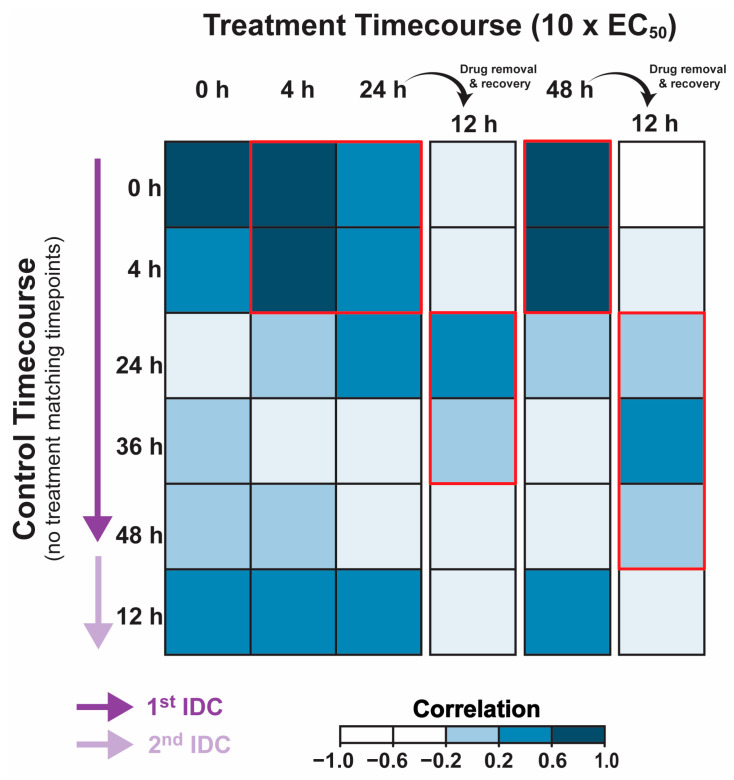
Transcriptional confirmation of CPW-B asexual cytostasis. Synchronized ring stage (10–12 hpi) *P. falciparum* strain 3D7 parasites were exposed to media supplemented with 500 nM CPW-B for 4, 24, or 48 h followed by RNA extraction. Total ring-stage parasite RNA was extracted just prior to the addition of CPW-B (0 h) to establish the transcriptome of the untreated population. RNA was also extracted after 24 or 48 h of drug exposure and 12 h of recovery in drug-free medium. All RNA was assayed via DNA microarray and total transcriptomes were compared to a previously published hourly asexual transcriptome. Correlation coefficients were calculated for each timepoint and displayed as a heatmap. Areas of highest correlation are highlighted by red outline.

The transcriptional program necessary for *P. falciparum* to progress through the asexual IDC has been extremely well characterized [29,30,31,32,33,34,35]. Therefore, any perturbation of the conserved transcriptome resulting from drug treatment and disruption of asexual progression should be apparent. Here, we utilized whole transcriptome abundance analysis to determine if there are gene expression changes that coincide with the treatment of *P. falciparum* strain 3D7 with CPW-B (10 × EC_50_). CPW-B was added to a highly synchronous ring stage culture and total RNA was collected just prior to the addition of CPW-B (0 h) and following 4, 24, and 48 h after drug exposure (Figure 5).

Additionally, as a control, CPW-B was washed out from a portion of the parasite culture following 24 or 48 h of drug treatment and grown for 12 additional hours in drug-free media. Each total RNA sample was then evaluated in comparison to a total RNA pool via DNA microarray to capture transcript abundance changes during and following treatment with CPW-B [36]. To determine if the cytostatic effects of CPW-B are supported by a corresponding “pausing” in the canonical transcriptional cascade, we first compared the total abundance of each transcript across the genome with the corresponding time post invasion from a previously published wild-type 3D7 transcriptome [37] (Supporting Information, Table S1). Remarkably, calculating the Pearson correlation coefficient revealed that the transcriptional program of the treated ring-stage parasites remains well-correlated to untreated ring-stage parasites regardless of treatment length (Figure 5 and Supporting Information Table S2: *corr* = 0.41–0.76). This suggests that CPW-B induces cytostatic “pausing” of the transcriptional program necessary to progress through the IDC coinciding with the lack of morphological progression of drug treated parasites. Interestingly, removal of CPW-B from the parasite after 24 and 48 h of incubation revealed that the treated parasites re-enter transcriptional progression during the 12 h of recovery in media with no drug as evidenced by the most positive correlation to the transcriptomic profile of 24–36 hpi trophozoites (Figure 5 and Supporting Information Table S2: *corr =* 0.25–0.33). Therefore, the transcriptional profiling of parasites during and after treatment with CPW-B confirms the cytostatic nature of this compound.

### 2.4. Cripowellin B Induced Cytostasis Is Not Reversed by Polyamine Supplementation

**Figure 6:**
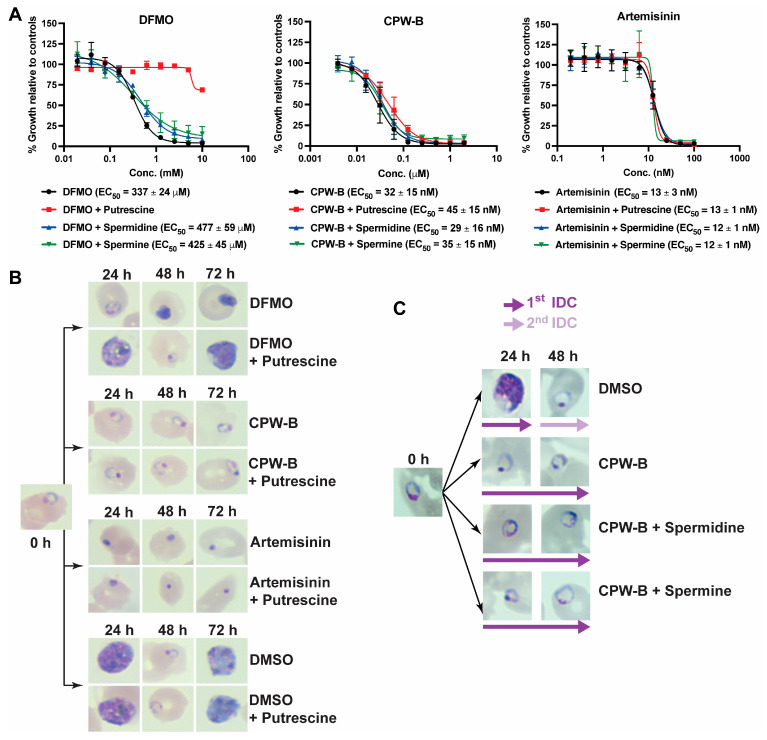
CPW-B-induced cytostasis is not reversed by polyamine supplementation. (**A**) Dose–response curves after 72 h continuous exposure in the presence or absence of 2 mM putrescine, 500 μM spermidine, or 30 μM spermine. The reported values represent averages and the SEM of three independent assays. (**B**) A representative Giemsa-stained thin blood smear is shown for cultures before treatments started (0 h) and at the indicated times for treated cultures with 2 mM DFMO, 500 nM CPW-B, 100 nM artemisinin, or 0.01% DMSO (vehicle) alone or supplemented with 2 mM putrescine. (**C**) CPW-B induced cytostasis is not reversed by spermidine or spermine supplementation. A representative Giemsa-stained thin blood smear is shown for cultures before treatments started (0 h) and at the indicated times for treated cultures with 0.01% DMSO (vehicle), 500 nM CPW-B alone or supplemented with 500 μM spermidine or 30 μM spermine.

Previous studies in *P. falciparum* provide evidence that cytostasis in the ring stage can be induced through the depletion of polyamines via inhibition of ornithine decarboxylase with the ornithine analog DL-α-difluromethylornithine (DFMO) [8]. Therefore, we hypothesized that CPW-B could be operating through a similar mechanism to DFMO by depleting the polyamine pool. To test this hypothesis, reversal of CPW-B growth inhibition by putrescine, spermidine, and spermine supplementation was assessed using the 72 h SYBR Green I assay as described in the method section. As expected, growth inhibition by DFMO was fully reversed only by supplementation with 2 mM putrescine (Figure 6A). However, reversal of CPW-B growth inhibition by putrescine, spermidine, and spermine supplementation was not observed (Figure 6A). Artemisinin was used as a negative control and its antimalarial activity was unaffected by polyamine supplementation (Figure 6A). In addition, 2 mM putrescine supplementation was confirmed to be non-toxic to parasites (Figure 6B). Giemsa-stained thin blood smears were performed every 24 h to assess the morphology of the parasites (Figure 6B). Altogether, our results suggest that putrescine supplementation did not reverse the cytostasis induced by CPW-B treatment in ring stage. Moreover, similar results were observed with the putrescine-derived polyamines spermine and spermidine (Figure 6C). Taken together, these results suggest that the cytostatic effect of CPW-B halting parasites in ring stage occurs through a cellular mechanism that differs from depletion of polyamines.

## 3. Materials and Methods

### 3.1. Chemicals

Cripowellin A (CPW-A) was previously purified by Presley and coworkers [23]. Cripowellin B (CPW-B, 90% purity) was a generous gift from Dr. Robert Velten from Bayer AG (Crop Science Division, Monheim, Germany). Compounds were reconstituted in DMSO. Artemisinin, putrescine, spermine, and spermidine were obtained from Sigma-Aldrich (St. Louis, MO, USA).

### 3.2. Plasmodium falciparum Culture

Parasites Dd2 and 3D7 strains were maintained in O^+^ human erythrocytes (Interstate Blood Bank, Memphis TN) at 5% hematocrit in RPMI 1640 media (GIBCO Life Technologies, Waltham, MA, USA) supplemented with 2 g/L glucose (Sigma-Aldrich, St. Louis, MO, USA), 2.3 g/L sodium bicarbonate (Sigma-Aldrich, St. Louis, MO, USA), 5.94 g/L HEPES (Sigma-Aldrich, St. Louis, MO, USA), 5 g/L Albumax I (GIBCO Life Technologies, Waltham, MA, USA), 50 mg/L hypoxanthine (Sigma-Aldrich, St. Louis, MO, USA), and 20 mg/L gentamicin (GIBCO Life Technologies, Waltham, MA, USA). Parasites were kept at 37 °C under reduced oxygen conditions (5% CO_2_, 5% O_2_, and 90% N_2_). Synchronous cultures in ring stage (>98%) were obtained by two consecutive cycles of 5% sorbitol treatment.

### 3.3. Plasmodium falciparum Growth Inhibition Assays

The in vitro effects of reported compounds were evaluated by the SYBR Green I assay as described previously [38]. Briefly, ring stage parasite cultures (100 μL per well, at 1% hematocrit and 1% parasitemia) were grown for 72 h in the presence of increasing concentrations of the inhibitor under reduced oxygen conditions (5% CO_2_, 5% O_2_, and 90% N_2_) at 37 °C. After 72 h in culture, growth was determined by DNA quantitation using SYBR Green I. The half-maximal effective concentration (EC_50_) values were calculated with GraphPad Prism (GraphPad Software, Inc., San Diego, CA, USA) using nonlinear regression curve fitting. The reported values represent averages of at least three independent experiments performed in triplicate using 10-point serial dilutions, with standard errors of the mean (SEM). The range for serial dilutions was adjusted accordingly for each test compound after the first screening to set the EC_50_ value in the middle of the concentration range. The final concentration of DMSO (vehicle) did not exceed 0.02%.

To assess the cytostatic effect of CPW-B, highly synchronous 6–12 hpi ring stage parasites 3D7 strain (3% parasitemia, 5% hematocrit) were treated with 500 nM CPW-B and bolus incubation times of 24, 48, 72, and 96 h. At each indicated time, CPW-B was washed out and parasites were maintained in drug free media for an additional 96 h. Parasitemia and intraerythrocytic stage progression were monitored over time by Giemsa-stained thin blood smears and light microscopy.

### 3.4. Plasmodium falciparum Transcriptomics and Analysis

To measure any changes in transcript abundance due to CPW-B, highly synchronous 10–12 hpi ring-stage *P. falciparum* strain 3D7 (7% parasitemia, 5% hematocrit) was exposed to 500 nM CPW-B. First, total ring-stage parasite RNA was extracted just prior to the addition of CPW-B (0 h) to establish the transcriptome of the untreated population. RNA was then collected following 4, 24, and 48 h of exposure to CPW-B. In addition, drug was removed from cultures that were exposed to CPW-B for 24 and 48 h by pelleting the entire culture in the centrifuge (5 min, 2000× *g*, 37 °C), removing the media supernatant and washing twice by resuspending in 50 mL regular medium followed by centrifugation. These “washed” parasites were placed back into standard culturing conditions and allowed to recover for 12 h prior to RNA extraction. Whole genome transcript abundance of each sample representing various treatment states was carried out by DNA microarray analysis as previously described [36]. Gene-level values were extracted and compared to the corresponding time point during the IDC from a previously published DNA microarray data set [37]. Comparison was performed using the *corrplot* R package (Version 0.92, https://github.com/taiyun/corrplot, accessed on 13 January 2023) with Pearson correlation coefficient displayed as a heatmap [39].

### 3.5. Reversal of P. falciparum Growth Inhibition by Polyamines Supplementation

To assess reversal of growth inhibition by putrescine, spermidine, and spermine, highly synchronous 6–12 hpi ring stage *P. falciparum* 3D7 strain cultures (100 μL per well, at 1% hematocrit and 1% parasitemia) were grown for 72 h in the presence of increasing concentrations of drug, and in the presence or absence of 2 mM putrescine, 500 μM spermidine, or 30 μM spermine. The reported values represent averages ± SEM of three independent experiments. Growth inhibition and recovery were assessed by SYBR Green I assay [38].

Morphology and stage development were monitored by light microscopy every 24 h under continuous drug exposure in the presence or absence of polyamines. Cultures starting in ring stage were treated with 2 mM DFMO, 500 nM CPW-B, 100 nM artemisinin, or 0.01% DMSO (vehicle) alone or supplemented with 2 mM putrescine. Reversal of growth inhibition by spermidine and spermine was assessed in cultures starting in ring stage and treated with: (1) 0.02% DMSO (vehicle), (2) 500 nM CPW-B, (3) 500 nM CPW-B and 500 μM spermidine, and (4) 500 nM CPW-B and 30 μM spermine. The concentration of each polyamine was selected based on previous reports [8,40].

## 4. Conclusions

Understanding the mechanisms that govern progression of the IDC in *P. falciparum* could reveal novel strategies for therapeutic intervention. Therapeutics targeting the ring stage of *P. falciparum* are of special interest to stop parasite progression to the mature forms that are sequestered by cytoadherence. In addition, the ring stage precedes gametocytogenesis thereby reducing or blocking transmission of the disease. Therefore, identifying the molecular target of the natural products CPW-A and B could potentially reveal the molecular mechanism behind ring stage development into the trophozoite stage, that in turn, could be used to rationally design or screen safer small molecules capable to target irreversibly this mechanism.

## Data Availability

The data presented in this study are available in this article and the Supplementary Materials.

## Supplementary Materials

The following supporting information can be downloaded at: https://www.mdpi.com/article/10.3390/molecules28062600/s1. Table S1: Whole genome DNA-microarray data for *P. falciparum* 3D7 strain that was exposed to 500 nM CPW-B and an untreated asexual time-course. All data are represented by timepoint and by gene. The numbers represent Log_2_(Cy3/Cy5 ratios) that have been centered by gene within a time course. Table S2: Pearson correlation coefficient calculation of the gene-by-gene data for each DNA microarray corresponding to individual experimental timepoints.
